# Microscale Thermophoresis as a Tool to Study Protein Interactions and Their Implication in Human Diseases

**DOI:** 10.3390/ijms23147672

**Published:** 2022-07-12

**Authors:** Romain Magnez, Christian Bailly, Xavier Thuru

**Affiliations:** 1 Univ. Lille, CNRS, Inserm, CHU Lille, UMR9020—UMR1277—Canther—Cancer Heterogeneity, Plasticity and Resistance to Therapies, 59000 Lille, France; 2 Oncowitan, Scientific Consulting Office, 59045 Lille, France; christian.bailly@oncowitan.com

**Keywords:** microscale thermophoresis, protein–protein interactions, biophysical methods, protein functions, protein antibodies, immune checkpoint, small molecules

## Abstract

The review highlights how protein–protein interactions (PPIs) have determining roles in most life processes and how interactions between protein partners are involved in various human diseases. The study of PPIs and binding interactions as well as their understanding, quantification and pharmacological regulation are crucial for therapeutic purposes. Diverse computational and analytical methods, combined with high-throughput screening (HTS), have been extensively used to characterize multiple types of PPIs, but these procedures are generally laborious, long and expensive. Rapid, robust and efficient alternative methods are proposed, including the use of Microscale Thermophoresis (MST), which has emerged as the technology of choice in drug discovery programs in recent years. This review summarizes selected case studies pertaining to the use of MST to detect therapeutically pertinent proteins and highlights the biological importance of binding interactions, implicated in various human diseases. The benefits and limitations of MST to study PPIs and to identify regulators are discussed.

## 1. Introduction

### 1.1. Implication of Proteins in Human Diseases

Proteins are made up of countless small units, called amino acids, linked together in chains of variable lengths, usually long chains of 50–500 units assembled into domains. Interactions between proteins (PPIs) and proteins with various partners (DNA, RNA) play diverse roles in biological processes and differ based on protein families, affinity and composition. Their association can be either permanent or transient [1]. Aberrant PPIs are associated with various diseases, including cancer, infectious diseases and neurodegenerative diseases to name only a few. Targeting PPIs is challenging but has become a mandatory strategy for the development of new drugs [2]. Due to their permanent nature, it is generally assumed that permanent PPIs are important for cellular function, whereas the pathological role (and targeting) of transient PPIs is still under debate [3]. There are many types of PPIs, with varied conformational and dynamic states, implicating monomeric proteins or protein complexes (homo and hetero oligomers) with varied stability features and located in varied cellular compartments. For example, proteins implicated in signal transduction exhibit usually short time effects that are not always easy to capture [4]. Another type of interaction is related to covalent interactions, which provide stronger association compared to non-covalent binding. Covalent modifications of proteins are encountered notably in the frame of post-translational modifications catalyzed by specific enzyme proteins [5]. The variability of PPIs is extremely large. It is important to take into account the type of complex we are dealing with when characterizing PPI interfaces, to adapt the best methods for detection and characterization of small molecule modulators. The primary/secondary structure of the proteins, their PPIs properties and their compartmentalization are important parameters to define the “druggability” of these proteins, i.e., the capacity of a given protein to be targeted with small molecules for therapeutic purposes. The design of new therapeutics frequently implicates the search for PPI modulators (inhibitors or activators) used to control specific pathophysiological signals and disease progression in general [6]. Here, we shall essentially concentrate our analysis on the field of cancer through the enumeration of anticancer drug development programs. These programs are frequently based in the notion of “cancer proteins” which have structural domains that exhibit a higher rate of infidelity than their non-cancer analogues. The differences make them more likely to interact with a wide variety of proteins, with altered protein partners or with different kinetics [7]. Proteins involved in cancer have many interaction partners and occupy a central position in the protein networks of cancer cells [7,8].

### 1.2. Overview of Protein Types and Their Relevance

Proteins are involved in almost all cellular activities. Protein synthesis is the creation of proteins by cells using DNA, RNA and various enzymes. It includes transcription, translation and other post-translational events, such as protein folding, modifications and proteolysis [9]. PPIs can lead to subtle changes in substrate binding by altering the kinetic properties of enzymes. They can also create a binding site for small molecules. They can inactivate a protein and change the specificity of a protein for its substrate with binding partners. They also can have a regulatory role. Their three-dimensional structure is directly linked to their function [10]. Proteins can be divided into sub-types such as structural proteins, contractile proteins, transport proteins, storage proteins, hormonal proteins, enzymes, protection proteins and antibodies [11,12,13]. PPI detection methods can be classified into three main types: in vitro, in vivo and in silico [14]. Proteins function by binding to another partner (proteins or molecules) and various technical approaches are available to study PPI and identify potential protein binders [15]. As mentioned previously, obligate PPIs are permanent. On the other hand, non-obligate interactions are transient, but some of them are also permanent (enzyme-inhibitor interactions). To summarize, PPI types can be either obligate or non-obligate and can be either transient or permanent [16].

### 1.3. Technical Approaches Used to Study PPIs

Targeting PPIs with a relatively flat interacting surface is often very challenging compared to isolated protein targets presenting well-defined binding pockets. However, methods have been adapted to these specific PPI architectures, leading to the identification of a large number of PPI inhibitors, discovered by high-throughput screening (HTS) and associated computational approaches. The methods are robust, but the identification of false positives is frequent [17]. Four investigational methods are commonly used to investigate PPIs and to identify their regulators: genetic (such as the use of yeast two-hybrid system), biochemical and biophysical methods (experimental approaches), and computational methods (including analysis of protein–protein docking interaction, for example). Some of the methodologies commonly used to study PPI are listed in Table 1. 

Most drug discovery programs use HTS to screen libraries of molecules presenting a large chemical or functional diversity. The approach is tedious and often expensive (successive runs of thousands of plated molecules consume a lot of time, reagents and data analysis time), but it is robust to generate early-stage modulators (hits) [18]. Another potential approach is the fragment-based drug discovery (FBDD) using small building blocks, but in that case, it is generally required to have access to the tridimensional structure of the protein target to build the final molecules from fragments. FBDD and HTS represent complementary approaches. The different approaches can be used to identify PPI inhibition targeted to the active site(s) of the protein (hot spots) and/or to allosteric sites [19]. Binding of a ligand to a hot spot competes with the protein partner of the PPI, resulting in disruption of function whereas allosteric PPI disruptors modify PPI affinity by binding to distal sites to the PPI surface [20].

**Table 1:** Most commonly used biochemical and biophysical methods to investigate PPIs.

Methods	Description	Ref.
Co-immunoprecipitation	Gold standard with endogenous proteins	[21]
Affinity electrophoresis	For binding constants	[22]
Phage display	HTS	[23]
Proximity ligation assay (PLA)	Immuno-histochemical method	[24]
Tandem affinity purification (TAP)	High-throughput identification	[25]
Surface plasmon resonance (SPR)	Label-free/immobilization required	[26]
Dynamic light scattering (DLS)	Screening/No immobilization or labeling	[27]
Bio-layer interferometry (BLI)	HTS/Label-free	[28]
Isothermal titration calorimetry (ITC)	Quantitative/Thermodynamics/No label or immobilization	[29]
Microscale thermophoresis (MST)	HTS/No immobilization/Can work in complex medium	[30]

### 1.4. Chemical Types of PPI Modulators

**Figure 1:**
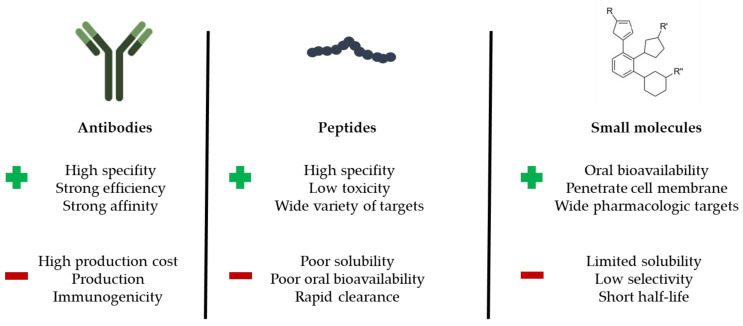
Comparison between antibodies, peptides and small molecules with main advantages and disadvantages as PPI modulators.

There are three main classes of PPI modulators: antibodies, peptides and small molecules. Monoclonal antibodies are attractive modulators and have high specificity. However, they have drawbacks, as they are not cell permeable or orally bioavailable and they present generally a high cost of manufacturing, leading to hard-to-produce, complex and expensive therapeutics [31]. Peptides also suffer from low bioavailability as well as a poor metabolic and proteolytic stability, and can induce immune reactions in some cases (as it is the case occasionally also with antibodies) [32]. However, one of the main advantages of peptides is the overall size (10–20 residues) and their defined conformation [33]. Small molecules have good bioavailability (in general) and are often produced at a much-reduced manufacturing cost compared to peptides and antibodies [34]. Pros and cons between PPI modulators are summarized in Figure 1. In all cases, the discovery of PPI modulators represents a solid approach to bring new chemical entities into clinical trials for the treatment of human diseases. This is notably the case for small molecules specifically designed for PPI implicated in oncogenesis and tumor development [35,36,37]).

Parenthetically, it is worth mentioning here an alternative approach to identify PPI modulators: the use of proteolysis-targeting chimeric molecules (PROTAC) which relies on proteasome-mediated proteolysis via the E3 ligase. The objective here is to engage the body’s own protein degradation system to selectively degrade pathogenic proteins to treat cancer and infectious diseases or other difficult-to-treat diseases rather than trying to physically block its interactions [38]. 

### 1.5. Quantifying Binding Interactions

The purpose of binding assays is to measure interactions between two molecules [39]. Those interactions can be a protein binding to another protein, a small molecule or a nucleic acid [39]. A dissociation constant (Kd) is an equilibrium constant that determines the affinity. Binding affinity refers to the strength of the binding interaction between a single biomolecule (such as a protein or DNA) and its ligand/binding partner (such as a drug or inhibitor). Binding affinity is usually measured and reported by the equilibrium dissociation constant, which is used to assess and prioritize the strength of biomolecule interactions. The lower the Kd value, the higher the binding affinity between the ligand and its target. The higher the Kd value, the weaker the attraction and binding of the target molecule to the ligand [40]. One simple way to define the dissociation constant is Kd = 1/Ka (association constant), with Ka dependent on the free energy of the interaction ΔG° = −RT lnKa, where R is the universal gas constant and T is temperature in Kelvins [41]. In order to generate a binding curve for a protein−ligand pair, a titration experiment can be carried out. One of the two partner is kept at constant concentration while the concentration of the second partner changes. The signal of the system will change with an increase or decrease depending on the technique used. Data can be plotted as fractions bound against the ligand concentration. The concentration of ligand bound to protein increases until it reaches saturation. The Kd value is the concentration at which half the ligand is bound [41]. Quantifying binding interactions provides a fundamental understanding of molecular biology and favors an improved disease treatment.

### 1.6. Microscale Thermophoresis 

Microscale thermophoresis is an immobilization-free technique for quantifying biomolecular interactions [30]. It measures the movement of molecules within a temperature gradient which induces changes in molecular properties of the studied molecules in terms of charge, size, hydration shell or conformation [42]. The MST technique is based on the directional movement of molecules along a temperature gradient. From a theoretical point of view, the directed movement of particles was originally described in 1856 [43]. Thermophoresis equations and mathematical background have been previously described in princeps publications [44,45]. From a practical point of view, the MST instrument records fluorescence of the sample with a focal infrared (IR) laser during and after the laser is turned on. Since the phenomenon of thermophoresis is diffusion limited, measurements are made within 30 s. The fluorescence traces contain different information on the binding event. The initial fluorescence has to be constant for every sample. Turning the IR-laser on results in a change in fluorescence intensity, called T-jump. This event relates to the temperature-dependent variation of fluorescence. The motion induces a concentration gradient of the fluorescent molecules. In the end, fluorescence intensity reaches a steady state where thermodiffusion is countered by mass diffusion [44]. When the laser is turned off, back diffusion occurs, lead by mass diffusion [44]. Affinity is quantified by monitoring the change in normalized fluorescence called Fnorm as a function of the concentration of the binding partner [44]. The Kd model used by MST describes a 1:1 stoichiometry interaction according to the law of mass action and allows to derive a formula for the fraction bound in case of a binding event [44]. The fraction bound is defined by the Kd and the concentration of the target molecule and depends on the ligand concentration [45].

**Figure 2:**
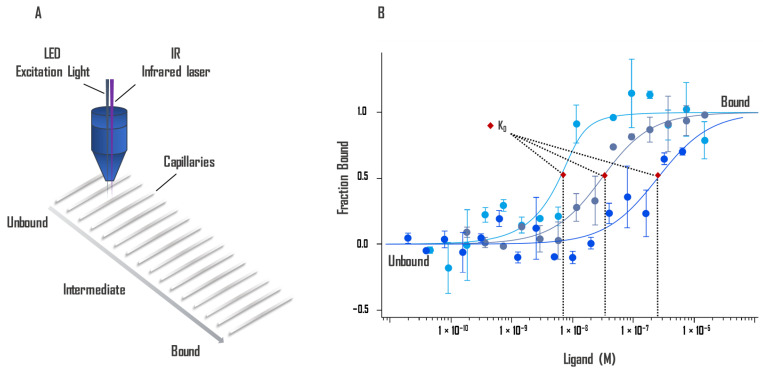
(**A**) Schematic representation of the optical system. Fluorescent molecules in the 16 capillaries are excited and the fluorescence detected by the same objective. An IR laser heats up locally, and thermophoresis of the fluorescent molecules across the temperature gradient is detected. (**B**) The intensity of fluorescence changes due to the movement of molecules away from the heated area differs when the ligand is bound. A binding curve can be established from difference of thermophoresis between the fluorescent molecules of both unbound and bound states against the ligand concentration. Binding constants Kd can be derived from binding curves. Graphs are represented as fraction bound against ligand concentration. Data represent three independent experiments and were fitted to a Kd binding model assuming a 1:1 binding stoichiometry.

The technique is extremely sensitive to any change in molecular properties, allowing precise analysis of binding events within a few microliters of solution for almost any molecule, such as protein-binding small molecules or ligands binding to liposomes or enzyme substrates. Since MST is a solution-based method, it avoids surface artifacts and immobilization protocols. MST is not restricted by the molecular weight ratio of partners involved in the binding like DLS and does not require a size change like SPR [46]. Besides the phenomenon of thermophoresis, the shape of the MST response is also influenced by the temperature-related intensity change (TRIC) [46]. TRIC is detected just after the IR laser is turned on, whereas thermophoresis is most noticeable at the later stage of signal collection. MST relies on the two effects, which are closely linked together [47]. An illustration of the technology is presented in Figure 2.

## 2. Case Studies

In the following subsections, we will refer to selected recent studies which have used MST to identify PPI modulators. The goal is to illustrate the interest of the technology with different types of molecules as well as various targets and signaling pathways to highlight the versatility of the methodology.

### 2.1. MST Applied to PROTAC Molecules

As mentioned above, a PROTAC is a bifunctional chimera composed of three parts: the targeting moiety, an E3 ubiquitin ligase binding moiety and a linker [48]. Once a PROTAC interacts with its target, the recruitment of the E3 ligase to the target protein results in ubiquitination and enables degradation via the proteasome. PROTACs have specific advantages such as low activity doses and selectivity that make them strong drug candidates. They also have limitations such as a difficult chemical synthesis and a potential toxicity [49]. 

#### 2.1.1. PROTAC-Induced Degradation of CREPT 

Pancreatic cancer is one of the hardest to treat and the deadliest type of cancers. Oncoprotein cell-cycle related and expression-elevated protein in tumors (CREPT) is highly expressed in pancreatic cancer and associated with poor disease-free survival. The targeting of CREPT with traditional chemotherapeutic drugs can lead to off-target effects and negative host responses. PROTAC molecules have been designed to effectively target CREPT and to suppress pancreatic tumor growth in a mouse model. MST helped to determine the affinity of the PROTACs for CREPT [50]. In that case, the PROTAC named PRTC was labeled with fluorescein (FITC), and purified glutathione S-transferase CREPT (GST-CREPT) protein was used. The results led to a Kd of 0.34 µM. PRTC-induced degradation of CREPT induced inhibition of pancreatic tumor growth in an experimental model. This example is illustrative because it refers to one of the first cases where MST was used to identify a PROTAC that interacts strongly with a protein crucial for the development of pancreatic cancer. Here, the MST technology has been efficient and very informative to discover and select the drug candidate. 

PROTACs have great potential in preclinical applications [51,52]. There are now several variants of the PROTAC approach, notably one using peptides as linkers (p-PROTAC) to increase specificity and to reduce off-target effects. Here again, MST has been useful to identify and select p-PROTAC [50,53]. One of the major advantages of using MST in this case was the fact that it avoided anthropogenic influence and increased authenticity with close to native conditions. 

#### 2.1.2. PROTAC-Induced Degradation of Brd4 

Bromodomain-containing protein 4 (Brd4) mediates the expression of genes involved in several human pathologies such as cancers, inflammatory diseases and acute heart failure [54]. Brd4 has been effectively targeted for degradation by different PROTACs, such as the compound MZ1 that induces reversible and selective removal of Brd4. The MZ1-Brd4 interaction was initially characterized using ITC, and a Kd value of 382 nM was determined [55]. Recently, an MST assay has been developed to investigate further this interaction due to its high sensitivity and the fact that it can be used in complex environment such as ternary complexes. In this case, a Kd value of 325 nM was calculated [56]. The agreement between the two technologies was excellent. The molecule has been further investigated to analyze ternary complexes formed with protein VCB (von Hippel−Lindau (VHL), Cullin and Elongin B/C, a trimeric subunit of an E3 ubiquitin ligase) and the MZ1-bromodomains complexes. This study using MST with three interaction partners led to the determination of Kd values of 6.7 nM and 12.1 nM, with proteins Brd3 and Brd4, respectively. This specific example highlights the benefit of using MST as a fast and sensitive method for PROTAC discovery. 

### 2.2. MST Applied to the PD-1/PD-L1 Immune Checkpoint

**Figure 3:**
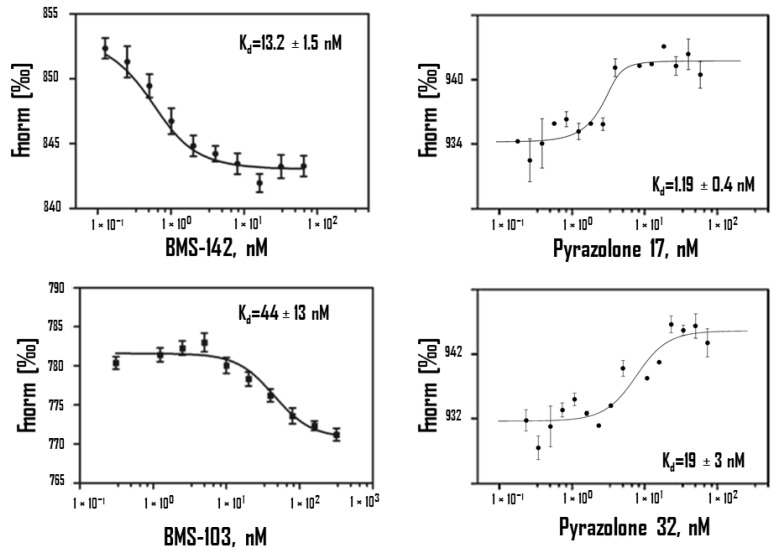
Binding of BMS-142, BMS-103, Pyrazolone 17, Pyrazolone 32 against human PD-L1 protein using MST. Binding of all four compounds to labeled PD-L1 resulted in a clear response in fluorescence signal, dependent on the concentration of the compound. Graphs are represented as Fnorm [‰] against ligand concentration. Data represent three independent experiments and were fitted to a Kd binding model assuming a 1:1 binding stoichiometry.

In oncology, the PD-1/PD-L1 immune checkpoint has been extensively studied over the past few years [57]. Protein programmed death-ligand 1 (PD-L1) is frequently over-expressed at the cell surface of various solid tumors and induces loss of immune function [58] when binding to programmed death 1 (PD-1), expressed at the surface of immune cells [59]. Monoclonal antibodies (mAb) targeting the interaction between PD-1 and PD-L1 are now extensively used to treat cancers [60,61]. The two components of the checkpoints can be targeted. There are currently ten anti-PD-1 and three anti-PD-L1 mAb registered [62]. The targeting of PD-L1 may be associated with less severe side effects compared to PD-1 [63,64]. Linked to the aforementioned downside of antibodies (Figure 1), other categories of PD-L1 binding molecules are being developed, in particular peptides and small molecules [65,66,67,68,69,70]. Different pharma companies, such as Bristol−Myers Squibb (BMS) and Aurigene (US), are intensely implicated in the design and development of small molecule PD-L1 binders, and for that they have used MST to characterize the PD-L1 binding capacity of the molecules [71]. Many academic groups are also implicated in the race to find new PD-L1 binders, including our group with the recent discovery of a category of pyrazolone-type molecules [72,73]. Several biophysical methods such as MST, SPR and ITC can be used to evaluate PD-L1 binding of such small molecules. In our case, we relied extensively on MST data to compare affinities of the pyrazolones. About 200 molecules have been designed and synthesized. MST was most helpful to identify the best hits, as indicated in Table 2. Although structurally different from BMS compounds, these small molecules have excellent affinity for PD-L1, within the nanomolar range (Table 2; Figure 3). MST has facilitated the identification of potent PD-L1 binders. Such small molecules may offer an alternative approach to mobilize the immune system to fight against cancers. 

Those small immunomodulators are still in early stages of development compared to one of the most promising PD-L1 targeting molecules, INCB086550, which has preliminary clinical data in patients showing increased immune activation and tumor growth control [74]. Therefore, small-molecule PD-L1 inhibitors may represent an effective alternative to restore antitumor immunity in patients.

### 2.3. MST in the Context of Gene Therapy

#### 2.3.1. CD19 CAR-T Cell Therapy

Chimeric antigen receptor (CAR) modified T-cell therapy based on antigen CD19 has shown promising results in the treatment of B-cell malignancies [75,76]. However, the use of murine CD19 CAR may lead to immune recognition in some patients and render the treatment ineffective [77,78]. A study has investigated whether the use of a humanized CD19 CAR would solve this issue. The authors used MST to measure affinities between CD19 and both murine or humanized CD19 CAR. Kd values obtained for CD19 mCAR and hsCAR were 509.4 ± 89.8 nM and 83.4 ± 12.2 nM, respectively [79]. Humanized CD19 CAR had a six-fold higher affinity for its human target. Since CARs are difficult to manufacture, using a very small quantity of protein was one of the major advantages of MST in this case. The broad applicability of the technique illustrated that humanized selective CD19 CAR is superior to its murine counterpart in terms of antigen-binding affinity. This is an interesting observation, which may lead in the future to improving therapies for patients with B-cell malignancies.

#### 2.3.2. CRISPR-Cas9-Based Gene Editing

Another example to underline the benefit of MST refers to the clustered regularly interspaced short palindromic repeats (CRISPR)/CRISPR-associated protein 9 (Cas9). CRISPR-Cas9 gene editing has shown promise to treat various diseases, including sickle cell anemia [80,81]. However, off-target gene editing has been reported for Cas9 proteins, such as *Streptococcus pyogenes* Cas9 (SpCas9) and *Francisella novicida* Cas9 (FnCas9) [82]. MST was used to show that FnCas9 has higher specificity for its intended target and low off-target binding effects [83,84]. FnCas9 and Spcas9 were tagged with a green fluorescent protein (GFP), and two DNA substrates were interrogated for the protein binding [85,86]. SpCas9 displayed a higher affinity (Kd = 49.6 nM and 10.9 nM, with the two DNA species) for both substrates than FnCas9 (Kd = 150.7 nM and 78.8 nM) [87]. However, FnCas9 did not bind to mismatched substrates whereas SpCas9 did with a Kd of 141.5 nM [87]. FnCas9 could be used to correct sickle cell mutations in patient-derived pluripotent stem cells [87]. This other example further illustrates the benefit of MST to compare protein affinities and to select protein therapeutics.

### 2.4. MST Applied to Coronavirus Infections

Coronaviruses (CoV) have been responsible for multiple epidemics this century [88]. The COVID-19 pandemic, caused by the severe acute respiratory syndrome coronavirus 2 (SARS-CoV-2), has led to a global health crisis. This emergency revealed the lack of CoV vaccines to prevent infection. It also revealed the urgent need for antiviral drugs to alleviate symptoms and shorten the duration of the disease. The lack of vaccines and antivirals is partly due to a poor understanding of the structural characteristics of the viral proteins that are involved in viral infection and replication [89]. Teams all over the world are using MST to study CoV since fast screening on large libraries can be performed [90]. Here we will highlight specific cases to illustrate the benefit of the methodology.

#### 2.4.1. Interactions between CoV Non-Structural Proteins 

The non-structural protein 15 (Nsp15) is an endoribonuclease that plays a major role in the life process of CoV and moderates evasion of host cell recognition by macrophages [91]. A biochemical and structural characterization of Nsp15 has been reported [92]. Using MST, purified Nsp15 was labeled and its interaction with various protein partners evaluated and quantified. The study confirmed that Nsp15 interacts directly with Nsp8 (Kd = 16.3 µM) and with the complex Nsp7/Nsp8 (Kd = 6.48 µM). This later protein complex is crucial for Nsp15 catalytic activity in RNA replication and transcription. In that case, MST contributed importantly to support the value of Nsp15 as a target for antiviral drug discovery.

#### 2.4.2. CoV Protein Nsp9 Binding to Single-Stranded DNA (ssDNA) 

Another non-structural protein which facilitates RNA synthesis and which is necessary for the CoV replication process is the non-structural protein 9 (Nsp9) [93]. Dimerization of Nsp9 is critical for viral replication, but the mechanism of formation or regulation of this process is still unclear [94]. Authors used wild-type (Wt) Nsp9 and specific Nsp9 mutants to identify which parts of the proteins are essential and involved in the binding with nucleic acid. MST allowed to quantify the interaction between Nsp9 mutants and ssDNA. Nsp9 proteins were diluted in serial dilution while ssDNA was labeled with the fluorescent cyanine dye Cy5. From the MST data, dose−response curves were recorded and analyzed to measure Kd values. The protein mutants were found to exhibit weaker affinities for the DNA substrate compared to the Wt protein [94]. Wt Nsp9 bound to the ssDNA with a Kd of 145 µM while mutant Nsp9-C59A exhibited a 2.7-fold binding reduction. The triple mutant Nsp9-G95E/G99E/G102E showed a 14-fold binding reduction and quadruple mutant Nsp9-C59A/G95E/G99E/G102E, a 36-fold binding reduction. This example illustrates the interest of the MST method to guide the identification of key amino acid residues that play an important role in DNA-binding proteins.

#### 2.4.3. Binding to CoV Glycoprotein S

Entry of CoV into cells is mediated by the transmembrane spike glycoprotein S. It forms a trimer that mediates binding to the host cell receptor and fusion of the viral and host cell membranes [95]. The structural characterization of this process has been technically challenging. MST was used to measure binding between stabilized pre-fusion trimer S from murine hepatitis virus (MHV) and the soluble host receptor [96]. Carcinoembryonic antigen-related cell adhesion molecule 1 (CEACAM1), a protein which mediates cell adhesion, was labeled with the RED-NHS labeling kit (Nanotemper Technologies) and titrated against a serial dilution of MHV S ectodomain protein. The MST binding curves led to the determination of Kd values in the nanomolar range (Kd = 48.5 nM). In this case, MST was instrumental to appreciate the metastable pre-fusion architecture of the spike glycoprotein and to identify interaction points essential to the stability of the protein complex [84].

### 2.5. MST Applied to Other Viruses

#### 2.5.1. Hepatitis C

Hepatitis C virus (HCV) affects more than 170 million people worldwide. HCV variability and the development of drug-resistant strains are leading the search for new antiviral agents [97]. Nonstructural protein 5A (N5SA) is a phosphoprotein that plays a key role in HCV infections [98]. MST data revealed that two clinically relevant inhibitors (BMS-790052 and AZD7295) bound to NS5A with high affinity (low-nM Kd) and inhibited RNA binding necessary for HCV replication [99]. In this particular case, MST was not only useful to compare different compounds and select leaders, but also to investigate their mode of action and in particular to show that the molecules do not affect the dimerization of NS5A [100,101].

#### 2.5.2. Influenza A

Influenza A virus spike protein binds to sialic acid on the cell membrane in a multivalent way [102]. Indeed, the protein has three binding sites allowing multivalent binding. Therefore, designing multivalent binders is a promising approach to prevent virus infection. MST has been used to validate a multivalent binder known to inhibit in vitro and in vivo virus infection [103]. Affinities of compounds were compared by MST to those obtained with the hemagglutination inhibition (HAI) assay. Both techniques led to similar binding affinities. The HAI value obtained was 1.3 ± 0.5 nM and the Kd value was 1.6 ± 0.2 nM using MST for the best binder (Qβ[Sia1]). MST was successfully used to guide the selection of highly potent compounds, exhibiting a nanomolar affinity for the protein target.

#### 2.5.3. HIV-1

Tripartite motif-containing (TRIM) proteins and especially TRIM5α play crucial roles in immunity and antiviral activity [104]. The human orthologue of TRIM5α (hsTRIM5α) fails to block infection by the human immunodeficiency virus 1 (HIV-1) both in vitro and in vivo [104]. At its C terminus, TRIM5α contains a B30.2 domain which can be exploited for binding studies. MST was used to measure binding affinity of Rhesus and human TRIM5α B30.2 domains to a series of HIV-1 capsid variants. MST measurements revealed a low Kd of 0.6 mM for the binding between rhTRIM5α B30.2 domain with the capsid dimer which differs from the capsid monomer (Kd = 1.6 mM). The results show that the interaction with the monomer is weaker compared to the dimer. The results also indicated that the HIV-1 capsid surface is pivotal for the binding of TRIM5α and its species-specific protection against infection in rhesus monkeys [105]. As the affinity expected was within the millimolar range, MST, thanks to its broad sensitivity scope, was a suitable tool to study low Kd’s between partners. The technology allowed to map the species-specific binding surface of the HIV-1 capsid for the first time.

## 3. Discussion

The MST technology can be applied to a broad range of biological systems, as illustrated above. The method has been instrumental to study protein–protein interactions applied to cancer, bacterial and viral pathologies, immune systems, gene therapy, drug design and many other biological and pharmacological situations. It is a robust and cost-effective method to determine ligand−protein affinities, whatever the nature of the protein receptor (monomer, multimeric complexes, heterogeneous assemblage, complex biological samples, etc.). All binding measurements are performed in solution, which provides a huge advantage compared to other technologies based on the surface-immobilization of the ligand (such as SPR). It is a method of choice to identify protein partners, complementary to other methods used to study PPIs [106]. In some cases, MST can also be used to investigate binding kinetics [107]. There are various methods to study PPIs (including ITC, SPR and capillary electrophoresis, for example), but MST is one of the most flexible and versatile to adapt to almost all situations, as illustrated here. In most cases, the binding conditions and the operational procedures can be quickly adapted, with small quantities of proteins solutions. Both purified protein solutions and complex biological mixtures (such as cell lysates, serum media, presence of detergents, liposomes, etc.) can be used without too many difficulties. The technology is highly adaptable and transposable to many types of proteins (from peptides to large protein complexes, from small molecule to antibodies). The methodology is well adapted to identify small molecule modulators of PPIs, offering a rapid screening approach. We have found the method particularly well-suited to characterize small molecule interrupters of immune checkpoints, be it the PD-1/PD-L1 checkpoint or other checkpoints such as TIM-3/galectin-9 [72,73,86]. It is clearly one of the best biophysical methods in early drug discovery [108]. MST is now a popular method to investigate PPIs and their regulations. There are sometimes difficulties to properly understand the thermodiffusion process in a biological context, but nevertheless the methodology is extremely useful and largely deployed [109]. Here, we essentially focused on PPIs, but the method is adaptable to other systems, such as aptamers, lipids and other types of biomolecular interactions [44,110,111,112,113,114]. The technology has been used for more than 15 years now and has contributed to a better understanding of many PPIs and the identification of many PPI modulators [115]. With no doubt, the use of this versatile and cost-effective method will continue to expand, with the continuous elucidation of PPIs and the identification of their many roles in human pathologies.

## 4. Conclusions

Microscale thermophoresis assays are routinely used to investigate protein–protein interactions and protein−ligand interactions and to guide the discovery of new modulators. The versatility and flexibility of the technology has been highlighted here, through various systems involved in oncology, viral diseases and immuno-inflammatory pathologies. MST is a convenient tool in drug discovery, most useful to rapidly evaluate and compare binding affinities for series of small molecules. This robust, easy-to-use, accurate and cost-effective technology is convenient to guide the selection of drug candidates. The use of this technology shall be encouraged. 

## Figures and Tables

**Table 2:** PD-L1 binding of selected small molecules evaluated by MST compared to SPR and ITC.

Compounds	MST Kd (nM)	SPR Kd (nM)	ITC Kd (nM)
BMSpep-57 *	19 ± 2	20 ± 2	/
BMS-103 *	44 ± 13	16 ± 2	**/**
BMS-142 *	13.2 ± 1.5	12 ± 2	**/**
Pyrazolone 11 **	83 ± 12	**/**	120
Pyrazolone 17 **	1.19 ± 0.4	**/**	/
Pyrazolone 32 **	19 ± 3	**/**	/

* Compounds from BMS, as described in [66]. ** Pyrazolone derivatives, recently designed and characterized [72,73].

## Data Availability

Not applicable.

## Abbreviations

BLI: bio-layer interferometry; Brd3: bromodomain-containing protein 3; Brd4: bromodomain-containing protein 4; CAR: chimeric antigen receptor; Cas9: CRISPR associated protein 9; CEACAM 1: carcinoembryonic antigen-related cell adhesion molecule 1; CoV: coronaviruses; CREPT: cell-cycle related and expression-elevated protein in tumors; CRISPR: clustered regularly interspaced short palindromic repeats; Cy5: cyanine 5; DLS: dynamic light scattering; DNA: deoxyribonucleic acid; FBDD: fragment-based drug discovery; FITC: fluorescein; FnCas9: francisella novicida Cas9; Fnorm: normalized fluorescence; GST: glutathione S-transferase; HAI: hemagglutination inhibition; HCV: hepatitis C virus; HIV-1: human immunodeficiency virus 1; hsTRIM5α: human orthologue of TRIM5α; HTS: high-throughput screening; ITC: isothermal titration calorimetry; Ka: association constant; Kd: dissociation constant; mAb: monoclonal antibodies; MHV: murine hepatitis virus; MST: microscale thermophoresis; NS5A: nonstructural protein 5A; Nsp15: non-structural protein 15; Nsp7: non-structural protein 7; Nsp8: non-structural protein 8; PD-1: programmed death 1; PD-L1: protein programmed death ligand; PLA: proximity ligation assay; PPIs: protein–protein interactions; PROTAC: proteolysis targeting chimera; RNA: ribonucleic acid; SARS: severe acute respiratory syndrome; SpCas9: streptococcus pyogenes Cas9; SPR: surface plasmon resonance; ssDNA: single-stranded DNA; TAP: tandem affinity purification; TIM-3: T-cell immunoglobulin and mucin containing protein-3; TRIC: temperature-related intensity change; TRIM: tripartite motif-containing; Wt: wild-type.
